# *Smilodon fatalis* Canine and Alveolus Junction Gap: Using MicroCT Scans and 3D Slicer

**DOI:** 10.1093/iob/obag014

**Published:** 2026-04-13

**Authors:** M S Haji-Sheikh, M J Haji-Sheikh, V L Naples

**Affiliations:** Department of Biological Sciences, Northern Illinois University, DeKalb, IL 60115 USA; Electrical Engineering, Northern Illinois University, DeKalb, IL 60115, USA; Department of Biological Sciences, Northern Illinois University, DeKalb, IL 60115 USA

## Abstract

The cranium of the sabertooth cat *Smilodon fatalis* from Rancho La Brea, now called La Brea Tar Pits and Museum (LBTPM), has been extensively studied; however, little research has been done on the placement and depth of the canines in the alveolus (tooth socket). During sample preparation, 110 years ago, canines often fell into the cleaning vats, so it is important to revisit and check these samples. Over the last 50 years, computerized tomography (CT) scans, including MicroCT and NanoCT scans, have moved from studying living humans to studying fossils. MicroCT scans can verify external observations such as misalignment of teeth. The reliance on 19th and 20th-century curations has always been fraught with complications. This study uses one of the largest collections of CT/MicroCT scans of this California sabertooth cat and demonstrates details that cannot be seen externally. A gap at the apical root would make the canine appear longer than in life. Results of our data set show that some of the specimens have improperly fitted canines, which can create errors in modelling. Finally, we demonstrate the use of 3D Slicer to virtually insert the canines and help determine whether a specific canine belongs to a specific cranium.

## Spanish

El cráneo del gato dientes de sable *Smilodon fatalis* de Rancho La Brea, ahora llamado La Brea Tar Pits and Museum (LBTPM), ha sido ampliamente estudiado; sin embargo, se ha investigado poco sobre la colocación y profundidad de los caninos en el alvéolo (cavidad dental). Durante la preparación de muestras, hace 110 años, los caninos a menudo caían en las cubas de limpieza, por lo que es importante revisar y comprobar estas muestras. En los últimos cincuenta años, las tomografías computarizadas (CT), incluidas las MicroCT y NanoCT, han pasado de estudiar humanos vivos a estudiar fósiles. Las MicroCT pueden verificar observaciones externas como la desalineación de los dientes. La dependencia de las curaciones de los siglos XIX y XX siempre ha estado plagada de complicaciones. Este estudio utiliza una de las mayores colecciones de escaneos CT/MicroCT de este gato dientes de sable de California y demuestra detalles que no pueden observarse externamente. Un espacio en la raíz apical haría que el canino pareciera más largo que en vida. Los resultados de nuestro conjunto de datos muestran que algunos de los especímenes tienen caninos mal ajustados, lo que puede generar errores en el modelado. Finalmente, demostramos el uso de 3D Slicer para insertar virtualmente los caninos y ayudar a determinar si un canino específico pertenece a un cráneo específico.

## German

Der Schädel der Säbelzahnkatze Smilodon fatalis aus Rancho La Brea, heute bekannt als La Brea Tar Pits and Museum (LBTPM), wurde ausführlich untersucht; jedoch wurde nur wenig Forschung zur Platzierung und Tiefe der Eckzähne im Alveolus (Zahnhöhle) betrieben. Während der Probenvorbereitung vor 110 Jahren fielen Eckzähne häufig in die Reinigungsbäder, weshalb es wichtig ist, diese Proben erneut zu überprüfen. In den letzten fünfzig Jahren haben sich computertomographische (CT-)Scans, einschließlich Mikro-CT- und Nano-CT-Scans, von der Untersuchung lebender Menschen hin zur Untersuchung von Fossilien entwickelt. Mikro-CT-Scans können äußere Beobachtungen wie Fehlstellungen der Zähne verifizieren. Die Abhängigkeit von Kurationen des 19. und 20. Jahrhunderts war stets mit Komplikationen verbunden. Diese Studie nutzt eine der größten Sammlungen von CT-/Mikro-CT-Scans dieser kalifornischen Säbelzahnkatze und zeigt Details, die äußerlich nicht sichtbar sind. Eine Lücke an der apikalen Wurzel würde den Eckzahn länger erscheinen lassen als im Leben. Die Ergebnisse unseres Datensatzes zeigen, dass einige der Exemplare falsch eingesetzte Eckzähne aufweisen, was zu Fehlern bei der Modellierung führen kann. Schließlich demonstrieren wir die Verwendung von 3D Slicer, um die Eckzähne virtuell einzusetzen und festzustellen, ob ein bestimmter Eckzahn zu einem bestimmten Schädel gehört.

## Portuguese (Brazil)

O crânio do tigre-dente-de-sabre *Smilodon fatalis* do Rancho La Brea, agora chamado La Brea Tar Pits and Museum (LBTPM), tem sido extensivamente estudado; no entanto, pouca pesquisa foi feita sobre o posicionamento e a profundidade dos caninos no alvéolo (cavidade do dente). Durante a preparação das amostras, há 110 anos, os caninos frequentemente caíam nos tanques de limpeza, por isso, é importante revisitar e verificar essas amostras. Ao longo dos últimos cinquenta anos, as tomografias computadorizadas (CT), incluindo MicroCT e NanoCT, passaram de estudos em humanos vivos para o estudo de fósseis. As varreduras de MicroCT conseguem verificar observações externas como o desalinhamento dos dentes. O embasamento em curadorias dos séculos 19 e 20 sempre foi repleto de complicações. Este estudo utiliza uma das maiores coleções de varreduras CT/MicroCT desse tigre-dente-de-sabre da Califórnia e demonstra detalhes que não podem ser vistos externamente. Uma lacuna na raiz apical faria o canino parecer mais longo do que era em vida. Os resultados do nosso conjunto de dados mostram que alguns dos espécimes possuem caninos encaixados de forma inadequada, o que pode criar erros na modelagem. Por fim, demonstramos o uso do 3D Slicer para inserir virtualmente os caninos e ajudar a determinar se um canino específico pertence a um crânio específico.

## Arabic

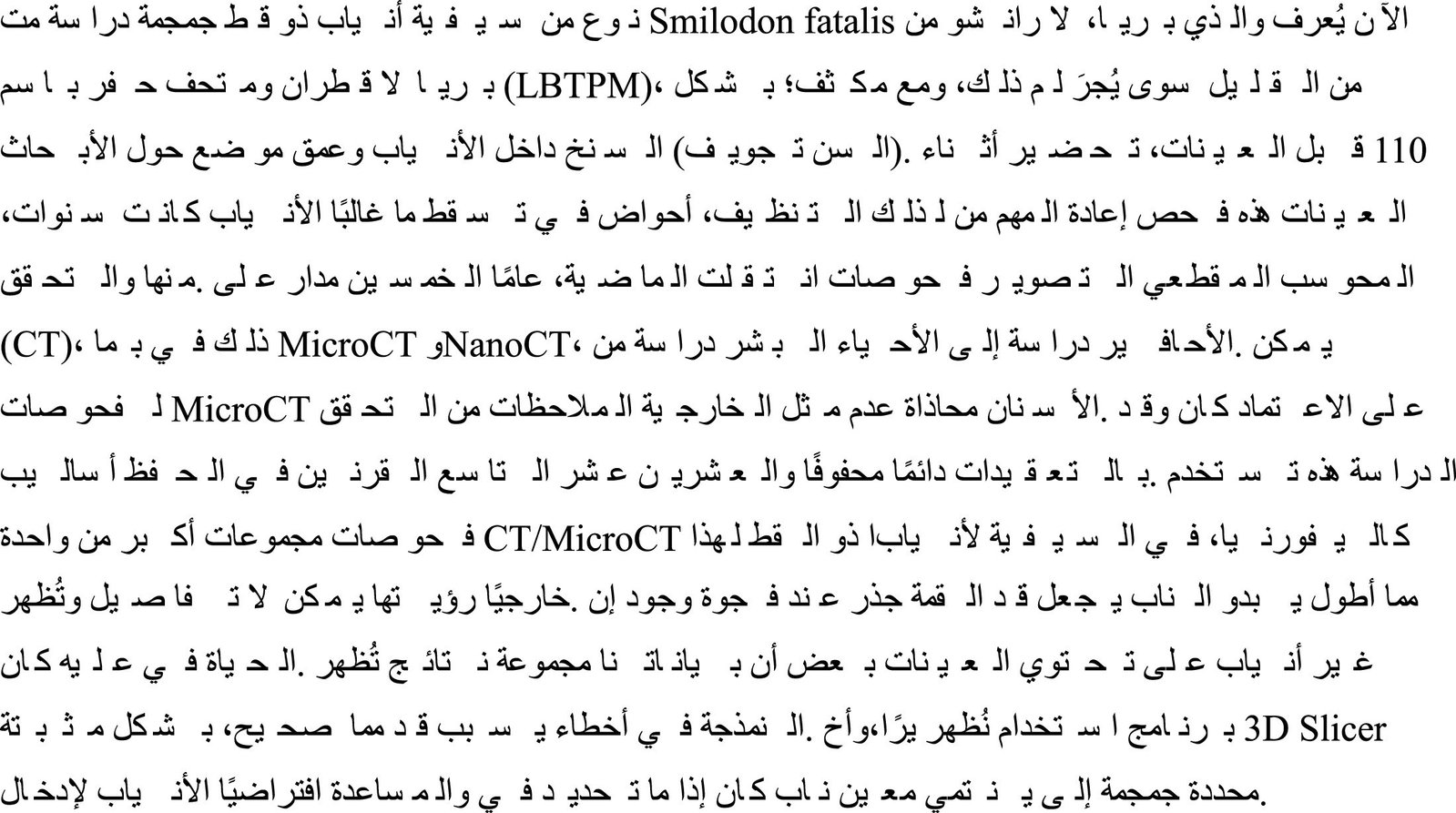

## Bengali

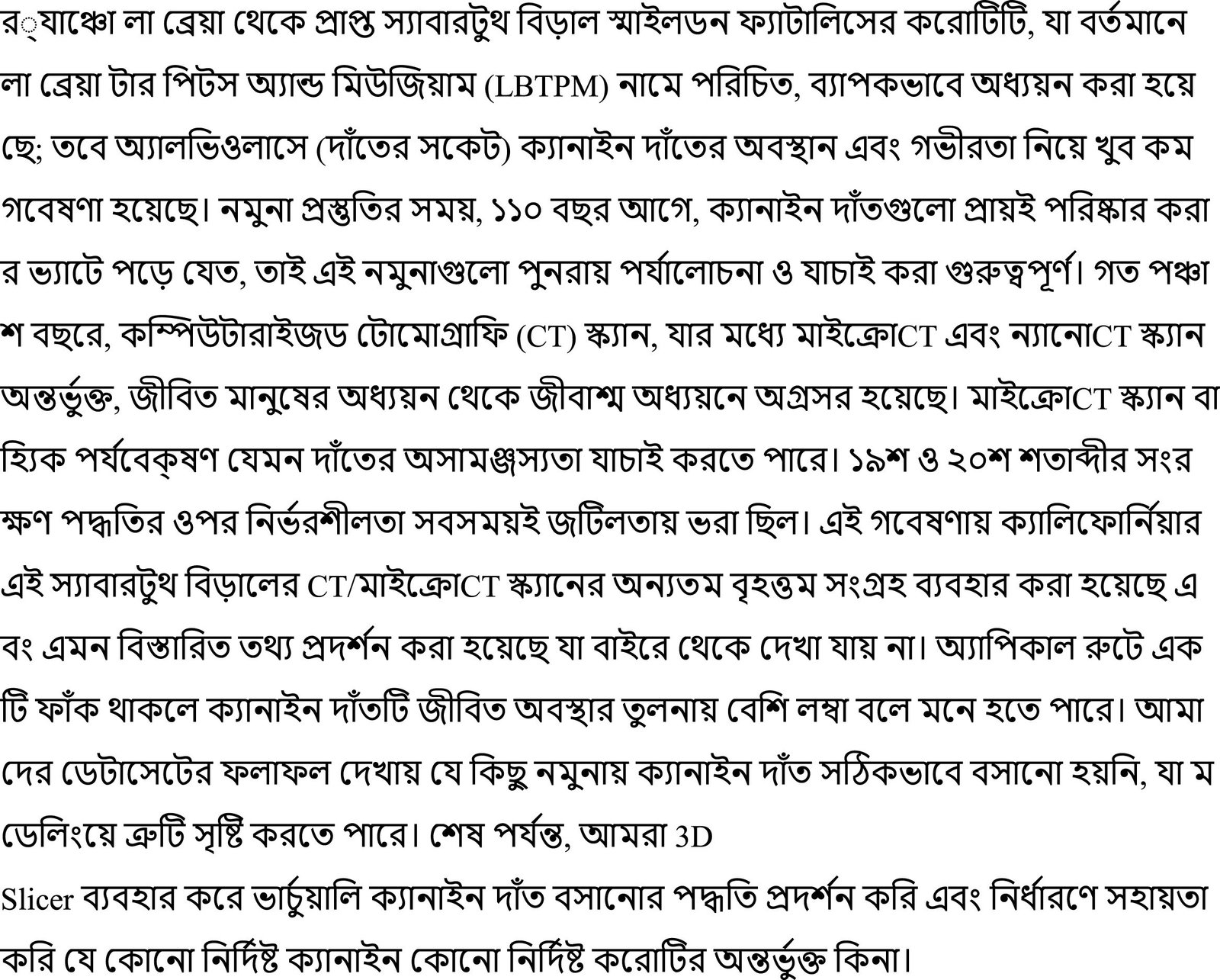

## Hindi

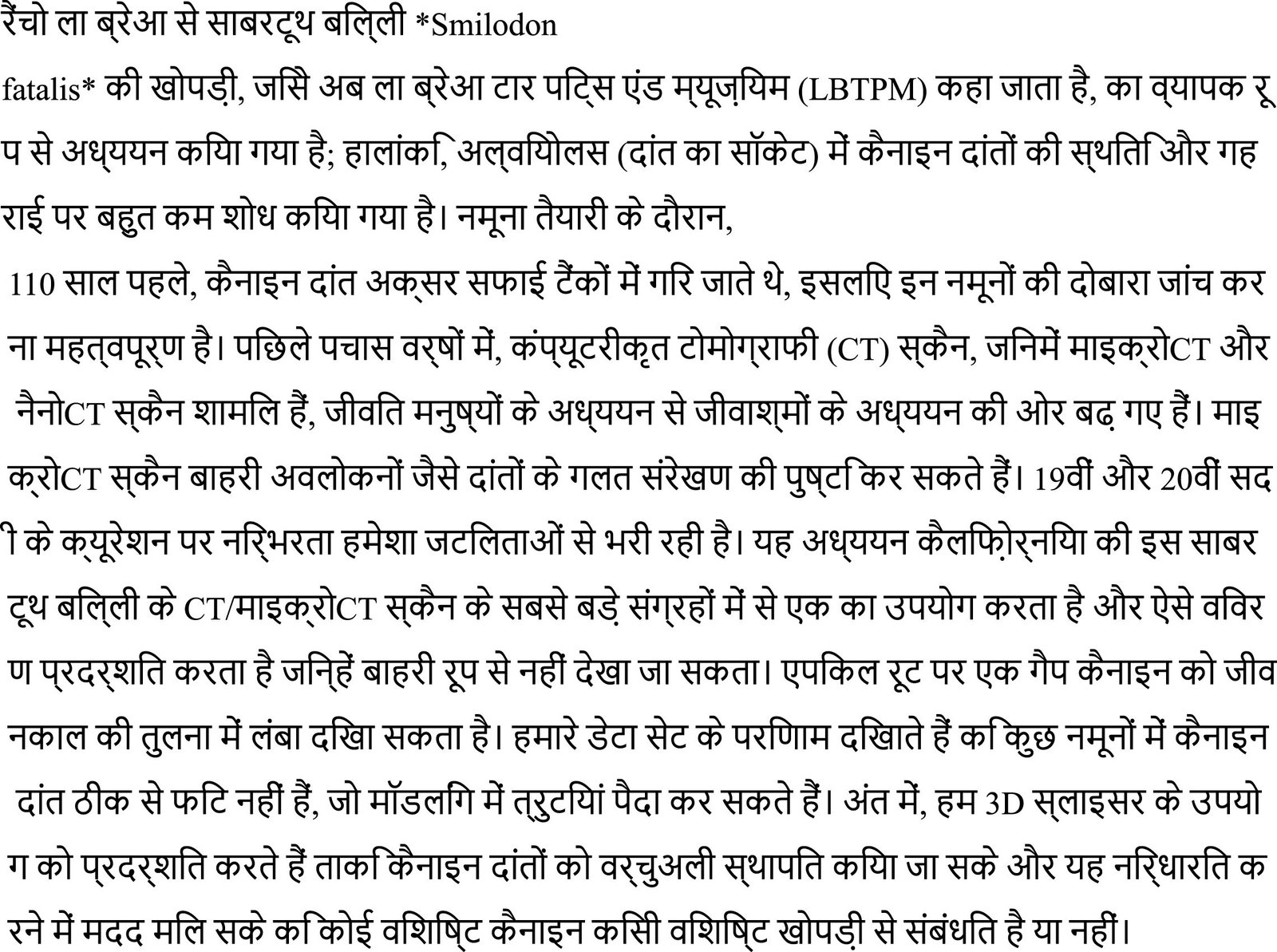

## Urdu

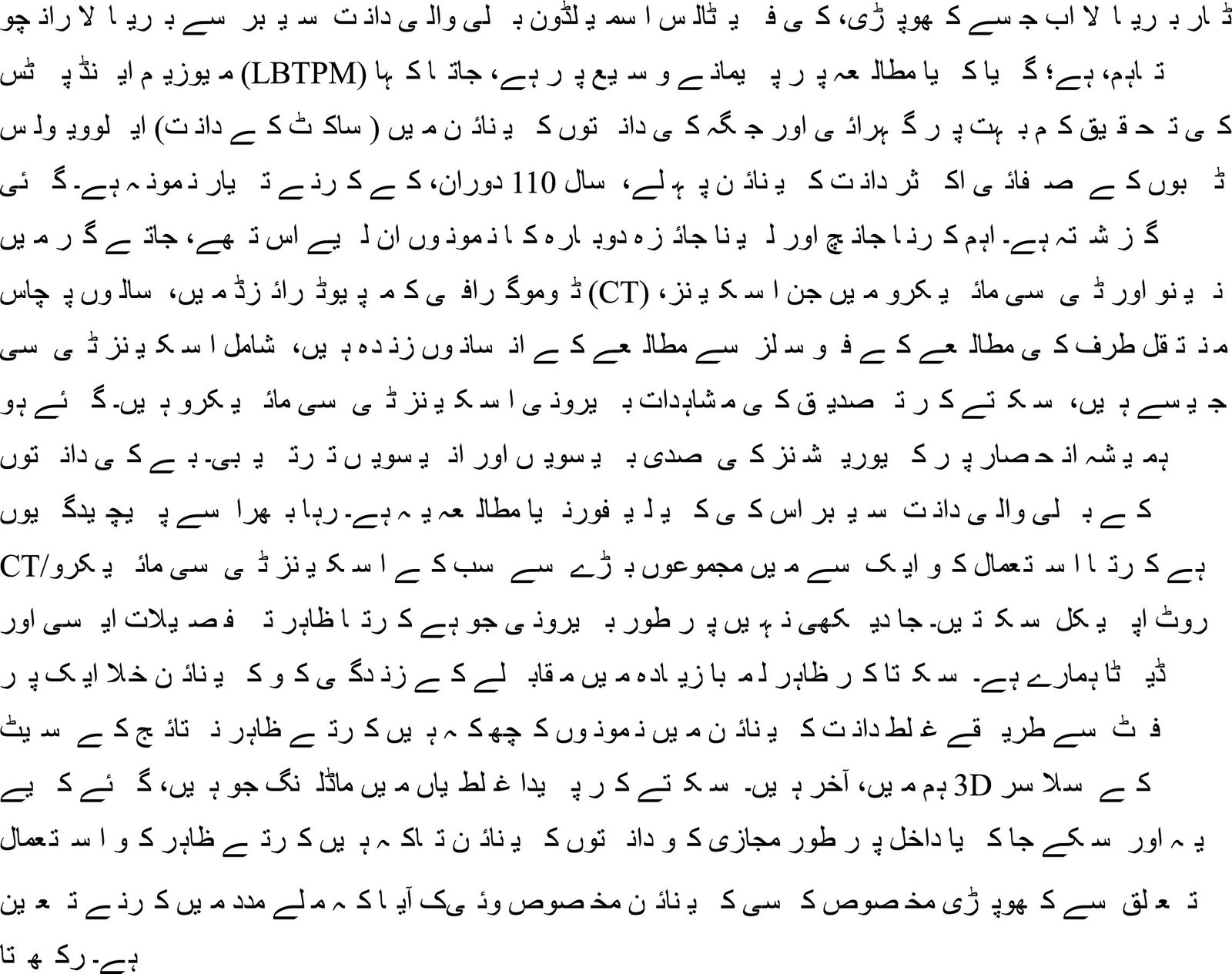

## Japanese

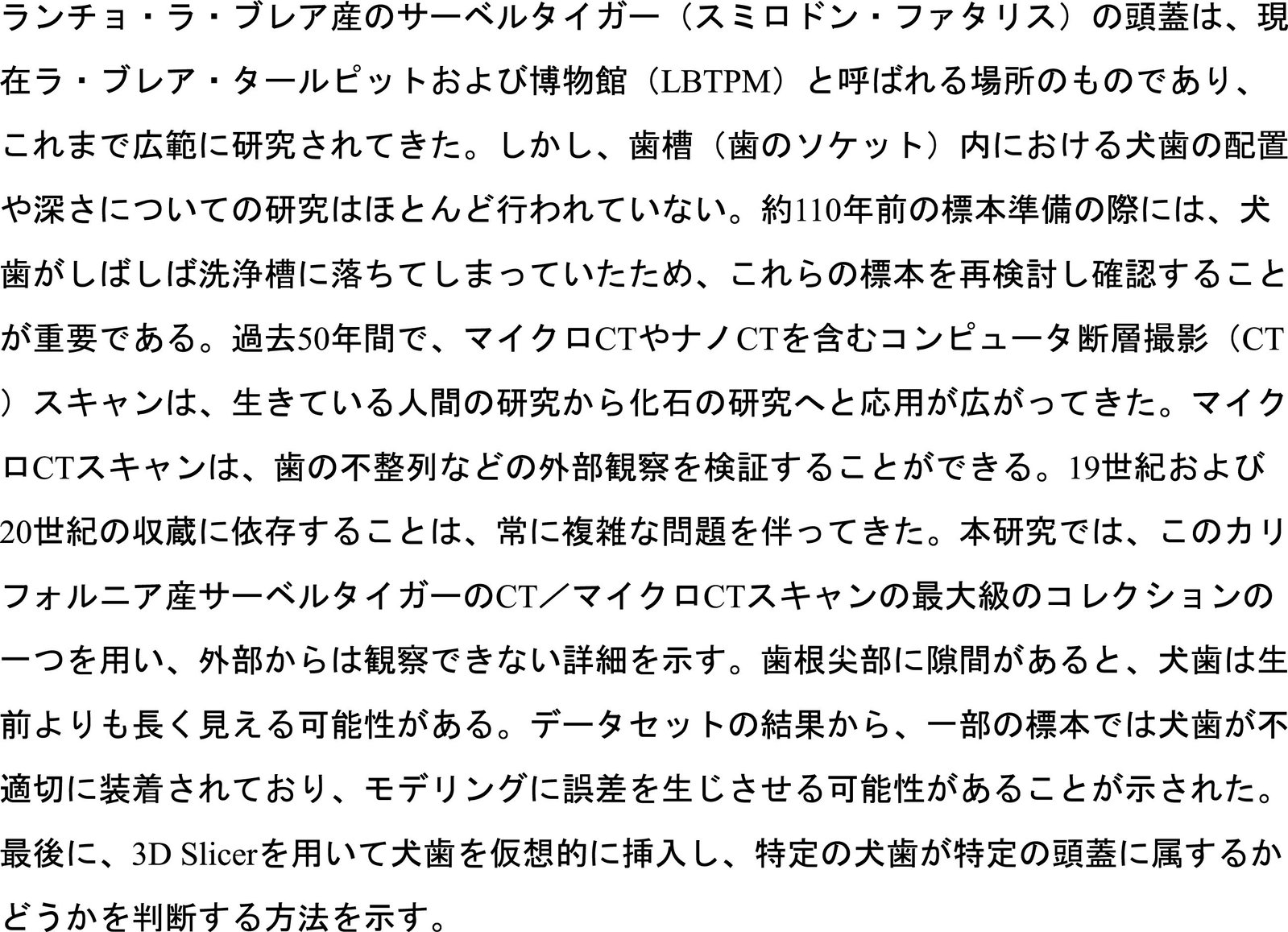

## French

Le crâne du chat à dents de sabre *Smilodon fatalis* provenant de Rancho La Brea, aujourd’hui appelé La Brea Tar Pits and Museum (LBTPM), a été largement étudié; cependant, peu de recherches ont été menées sur le positionnement et la profondeur des canines dans l’alvéole (cavité dentaire). Lors de la préparation des échantillons, il y a 110 ans, les canines tombaient souvent dans les cuves de nettoyage, il est donc important de revisiter et de vérifier ces spécimens. Au cours des cinquante dernières années, les scans de tomographie informatisée (CT), y compris les scans MicroCT et NanoCT, sont passés de l’étude des humains vivants à celle des fossiles. Les scans MicroCT peuvent vérifier des observations externes telles que le mauvais alignement des dents. La dépendance aux pratiques de conservation des XIXe et XXe siècles a toujours été entachée de complications. Cette étude utilise l’une des plus grandes collections de scans CT/MicroCT de ce chat à dents de sabre californien et met en évidence des détails invisibles extérieurement. Un espace au niveau de la racine apicale ferait paraître la canine plus longue qu’elle ne l’était de son vivant. Les résultats de notre ensemble de données montrent que certains spécimens possèdent des canines mal ajustées, ce qui peut entraîner des erreurs dans la modélisation. Enfin, nous démontrons l’utilisation de 3D Slicer pour insérer virtuellement les canines et aider à déterminer si une canine spécifique appartient à un crâne spécifique.

## Russian

Череп саблезубой кошки Smilodon fatalis из Ранчо Ла-Бреа, ныне называемого La Brea Tar Pits and Museum (LBTPM), был тщательно изучен; однако мало исследований было посвящено положению и глубине клыков в альвеоле (зубной лунке). Во время подготовки образцов 110 лет назад клыки часто выпадали в очистительные ванны, поэтому важно вновь пересмотреть и проверить эти образцы. За последние пятьдесят лет компьютерная томография (КТ), включая MicroCT и NanoCT, перешла от изучения живых людей к исследованию ископаемых. Сканирование MicroCT может подтверждать внешние наблюдения, такие как смещение зубов. Опора на коллекции XIX и XX веков всегда была сопряжена с трудностями. В данном исследовании используется одна из крупнейших коллекций КТ/MicroCT-сканов этой калифорнийской саблезубой кошки и демонстрируются детали, которые невозможно увидеть снаружи. Промежуток у апикального корня может делать клык визуально длиннее, чем он был при жизни. Результаты нашего набора данных показывают, что у некоторых образцов клыки установлены неправильно, что может приводить к ошибкам при моделировании. Наконец, мы демонстрируем использование 3D Slicer для виртуальной вставки клыков и помощи в определении того, принадлежит ли конкретный клык конкретному черепу.

## Chinese

來自 Rancho La Brea(現稱 La Brea 沥青坑與博物館, LBTPM)的劍齒虎 Smilodon fatalis 頭顱骨已被廣泛研究;然而, 對於犬齒在牙槽(牙齒插座)中的位置和深度的研究卻很少。在樣本準備過程中, 110 年前, 犬齒經常掉入清洗槽, 因此重新檢查這些樣本非常重要。在過去五十年中, 電腦斷層掃描(CT 掃描), 包括 MicroCT 和 NanoCT 掃描, 已從研究活人擴展到研究化石。MicroCT 掃描可以驗證如牙齒錯位等外部觀察結果。對於 19 世紀和 20 世紀的標本整理, 一直存在諸多複雜問題。本研究使用加州劍齒虎最大型的 CT/MicroCT 掃描收藏之一, 展示了無法從外部觀察到的細節。牙根尖端的空隙會使犬齒看起來比實際生活中更長。我們的數據集結果顯示, 一些標本的犬齒安裝不當, 可能會在建模中造成錯誤。最後, 我們展示了使用 3D Slicer 虛擬插入犬齒的方法, 幫助判斷特定犬齒是否屬於特定頭顱骨。

## Hebrew

תַקצִיר

הגולגולת של חתול החרב Smilodon fatalis מ-Rancho La Brea, הנקראת כיום La Brea Tar Pits and Museum (LBTPM), נחקרה בהרחבה; עם זאת, מחקר קטן נעשה על המיקום והעומק של הניבים במכתש (שקע השן). במהלך הכנת הדגימות, לפני 110 שנה, נפלו לעתים קרובות כלבים לתוך בורות הניקוי, ולכן חשוב לחזור ולבדוק דגימות אלו. במהלך חמישים השנים האחרונות, סריקות טומוגרפיה ממוחשבת (CT), כולל סריקות MicroCT ו-NanoCT, עברו מחקר בני אדם חיים לחקר מאובנים. סריקות MicroCT יכולות לאמת תצפיות חיצוניות כגון חוסר יישור שיניים. ההסתמכות על אוצרות מהמאה ה-19 וה-20 תמיד הייתה רצופה בסיבוכים. מחקר זה משתמש באחד האוספים הגדולים ביותר של סריקות CT/MicroCT של חתול חרב זה בקליפורניה ומדגים פרטים שלא ניתן לראות מבחוץ. פער בשורש הקודקוד יגרום לכלב להיראות ארוך יותר מאשר בחיים. תוצאות מערך הנתונים שלנו מראות שלחלק מהדגימות יש ניבים מותאמים בצורה לא נכונה, מה שעלול ליצור שגיאות בדוגמנות. לבסוף, אנו מדגימים את השימוש ב-3D Slicer כדי להכניס את הניבים באופן וירטואלי ולעזור לקבוע אם כלב ספציפי שייך לגולגולת מסוימת.

## taktzir

hagulgolet shel chatol hachrev Smilodon fatalis me-Rancho La Brea, hanikret kiyum La Brea Tar Pits and Museum (LBTPM), nechakra beharchava; im zot, mechker ktan nease al hamikum veha’omek shel hanivim bemachtash (shka hashen). bemehalech haknet hadgimot, lefani 110 shena, niflu le’atim krovot klavim letuch burot hanikui, valken hashuv lechzor velbeduk dgimot alu. bemehalech chmishim hashanim ha’achronot, sarikot tomografia memuchshevet (CT), kolel sarikot MicroCT v-NanoCT, ivru mechker bani adam hiyim lechker meuvanim. sarikot MicroCT yecholot le’amet tzpiot hitzoniot kegon hoser yishur shini’im. ha’histamchot al otzrot mehama’a ha-19 veh-20 tmid hayata retzufa besibuchim. mechker ze mishtamesh be’achad haosfim hagdulim bioter shel sarikot CT/MicroCT shel chatol chrev ze baklifornia umadgim partim shale nitan lir’ot mivchutz. pa’ar beshursh hakodkud yigrom lechlav lehiraot aroch yoter me’asher bechi’im. totsaot ma’arech hanatonim shlanu mir’ot shlachlak mehadgimot yesh nivim mutamim betzura le nichona, meh shalul litzur shgi’ot bedugmanot. lebasof, anu midgimim et hashimush be-3D Slicer kadi lehaknis et hanivim be’ufan virtuali velazor lekvua am klav siftzifi shayech legulgolet mesuyamet.

## Introduction

Leidy identified the sabretooth cat, *Smilodon fatalis*, in 1869 (Leidy 1869). This apex predator lived in North America from the Pleistocene to the early Holocene epoch and became extinct approximately 10,000–12,000 years ago. This felid, with its elongated canines, and no living analog, has perennially fascinated scientists and the public. However, due to taphonomy, early preparatory techniques (discussed below), and the simple, single-rooted anatomy of canines, the provenance of canines in *S. fatalis* specimens can be problematic. The teeth may have slipped from their original life positions or might be from another specimen entirely. This information is crucial for research; however, it is not often included in studies, as it is best determined by examination of internal anatomy. Here, we use MicroCT to examine the canine alveolus junction in 10 *S. fatalis* specimens to assess the provenance of their iconic sabers.

The Rancho La Brea Tar Pits and Museum, now called La Brea Tar Pits and Museum (LBTPM), with the world’s most extensive and invaluable collection of *S. fatalis* specimens, is a cornerstone in our understanding of this sabretooth. Merriam and Stock first described this felid in 1932 (Merriam and Stock 1932). During this time, the specimens were cleaned by immersion, typically using kerosene as a solvent, followed by mechanical and additional chemical treatments to clean the bone surfaces. Shaw (1982) describes how the original method was done: “Asphalt-encrusted bones were soaked in vats of kerosene heated over open fires until the asphalt dissolved or was softened sufficiently to be removed by hand during repeated submergences in the solvent.” Once this cleaning was completed, the cranial and postcranial measurements were taken directly from the available skeletal elements. Later samples were cleaned using a more modern technique outlined by Shaw (1982), which used vapor degreasing. The importance of understanding the basic handling of fossils prior to analysis cannot be underestimated. In Merriam and Stock (1932), on page 57, is a comment about the loss of a specimen’s deciduous canines: “SKULL No. 2001—9—Deciduous canines are absent but probably dropped from sockets during the period of preservation. . .” The probability of specimen disarticulation during cleaning in the early days of Rancho La Brea is not zero; therefore, it is important to verify the specimen’s condition before accepting the data.

The cranium of *S. fatalis* has also been extensively studied to determine bite, bite strength, gape, killing methods, hunting, and feeding styles. (Miller 1969; Feranec 2004; Anderson et al. 2011; Meachen 2012; Brown 2014). Moreover, the canine teeth of *Smilodon fatalis* have been a subject of scientific fascination. These canines have been studied by authors for almost a century by Merriam and Stock (1932), Emerson and Radinsky (1980), Deutsch et al. (2025), Pollock et al. (2025), Shelbourne and Lautenschlager (2025), Tseng (2025), and others. However, these studies were based on external craniodental anatomy, they do not discuss the canine being fully seated in the alveolar socket. The last time the growth and shape/structure of the alveoli was approached was by Akersten (1985). Unfortunately, there is no supporting data about the alveolus in his paper.

In some cases, museum information is available for specimens regarding canine provenance. For example, specimen FMNH_P_12418 from the Field Museum of Natural History (FMNH) can be associated with personal communications from Simpson (2025) stating in part, FMNH_P_12418 is part of the mounted skeleton on exhibit in Evolving Planet. . . The database says it has only one real saber, which has a different catalogue number than the composite mount, which means it has been inserted into the alveolus. . .” However, such information can be missed [FMNH_P_12418 has been stated to have complete upper canine roots in Wroe et al. (2013)].

While various methods traditionally have been employed to study these canines, including measuring, drawing, and analyzing their structure and function using methods such as finite element analysis for bone stress calculations. The use of a significant sample size of computerized tomography (CT) to focus on the placement of the canines fully in the alveolus is a novel approach.

Here, we evaluate MicroCT scans of 10 *S. fatalis* crania (both complete and partial skulls). We also demonstrate the flexibility of 3D Slicer’s features in aligning multiple MicroCT scans to determine if elements, in this case the canines, which have been disassociated, are actually associated. Based on previous radiograph exams of the canine alveolar socket seating in extant carnivorans, external examination of LBTPM skulls, and museum database information (e.g., missing canine provenance in the specimen records). We had reason to believe that not all the specimens in our dataset had correctly seated and/or associated canines. External observations and measurements would not determine the correct placement of the canines. Hence, we designed this using MicroCT scans to examine the internal anatomy and test this. Additionally, we present new images with a specific focus on the area where the canine teeth are inserted into the alveoli.

## Materials and methods

### Specimen source

All specimens used in this study were originally discovered and recovered from LBTPM. In total, 10 specimens were analyzed from three different collections: The FMNH, LBTPM, a Division of the Natural History Museum of Los Angeles County (LACM), American Museum of Natural History in New York (AMNH). The two specimens from LACM (LACM 2001–3 and LACM: RLB:37376) were available on request and are housed on MorphoSource. Specimen FAM 14349 from the AMNH in New York required obtaining express permission from the curators prior to downloading from MorphoSource. In the end, we decided to rescan the third specimen LACM 2001–3 with a much higher resolution than was previously performed. Table 1 shows the specimen ID, the institution which holds the specimen, and the condition of the specimen.

**Table 1:** Shows the specimen ID, the institution which holds the specimen, and the condition of the specimen.

ID Number	Institution/Collection	CT Scan System/Scan resolution	Specimen condition
FMNH_P_12417(Fig. 2)	Field Museum of Natural History	Phoenix v|tome|x s 240 MicroCT xyz 132.852 µm. 150kV, 80µA, 0.1 mm Cu filter, 500ms exposure, 2000 projections, 3 average frames and 1 skipped frame.	Mostly complete specimen. On floor display at FMNH. Outward view has 2 canines and museum notes do not mention any special conditions.
LACMP23-7550(Figs 3, 4)	Natural History Museum of LA County	Nikon Metrology XT H 225 ST xyz 122.278 µm. 90kV, 120µA, 1000ms exposure, 2000 projections	Mostly complete some pit wear. New specimen with most of two canines. First complete specimen from Project 23 was discovered in 2008.
LACM 2001–2(Figs 1, 5, 6, 7)	Natural History Museum of LA County	Nikon Metrology XT H 225 ST xyz 122.278 µm. 90kV, 150µA, 0.1 mm Cu filter, 1000 ms exposure, 2000 projections	Mostly complete -one broken canine Used by Merriam and Stock (1932), Akersten (1985), and Deutsch et al. (2025)
LACM 2001–3	Natural History Museum of LA County	Nikon Metrology XT H 225 ST xyz 122.278 µm. 90kV, 120µA, 0.1 mm Cu filter, 1000 ms exposure, 2000 projections,	Mostly complete -one broken canine
FMNH_PM_3671	Field Museum of Natural History	Phoenix v|tome|x s 240 MicroCT xyz 132.852 µm. 150kV, 80µA, 0.1 mm Cu filter, 500 ms exposure, 2000 projections, 3 average frames and 1 skipped frame.	Missing premaxillary—Used by Radinsky (1975) for his endocast work
LACM:RLB:R37376(Fig. 8)	Natural History Museum of LA County	Varian Medical Systems (Bio-Imaging Research, Inc) ACTIS scanner with GE Titan X-ray source, xy 210 µm z 500 µm,420 kV, 1.8 mA, 629 projections	Mostly complete. One canine missing one with 30 mm crown left. Used by Wroe et al. (2013) in modeling.
FMNH_P_12407(Not shown)	Field Museum of Natural History	Phoenix v|tome|x s 240 MicroCT xyz 108.946 µm. 150kV, 80µA, 0.1 mm Cu filter, 500ms exposure, 2000 projections, 3 average frames and 1 skipped frame.	Right maxilla only—mostly complete alveolus.
FMNH_P_12463(Not shown)	Field Museum of Natural History	Phoenix v|tome|x s 240 MicroCT xyz 78.832 µm. 150kV, 80µA, 0.1 mm Cu filter, 500 ms exposure, 2000 projections, 3 average frames and 1 skipped frame.	Nasal area, premaxilla, and parts of alveolus missing.
FAM 14349(Not shown)	American Museum of Natural History	General Electric Phoenix v|tome|x Anterior 1/3 xyz 142 µm, Posterior 2/3 xyz 284 µm	Mostly complete. Used by Jack Tseng for modeling. Both canines are broken
LACMHC 6870(Fig. 9)	Natural History Museum of LA County	Nikon Metrology XT H 225 ST xyz 122.278 µm. 90kV, 150µA, 0.1 mm Cu filter, 1000 ms exposure, 2000 projections	Mostly complete. Disarticulated canines. Collagen loss and delamination. Sutures appear to be reopened.

Table 1 shows the specimen ID, the institution which holds the specimen, and the condition of the specimen.

### Micro computed tomography

Table 1 shows the source of specimens used in this study. Specimens from paleobiology collections of the FMNH were scanned at the University of Chicago PaleoCT Lab using state-of-the-art research CT systems, a Phoenix v|tome|x s 240 micro computed tomography (MicroCT), with voxel resolution in the 100–130 µm range. The four in-house skulls from the LACM collection were scanned at the Medical Molecular Imaging Center of the University of Southern California (USC) Department of Radiology, using a Nikon Metrology XT H 225 ST system at a resolution of 0.122 mm. Additionally, the canines from LACMH 6870 were scanned at an ultra-high resolution of 0.0583 mm with a GE Nanotom Phoenix M. This scan is a NanoCT scan. The data, from LACM_rlb_R37376, uploaded to MorphoSource, was from 2003 and had a resolution of 210 µm in *x* and *y* and 0.5 µm in *z*. The specimen from the AMNH, due to the limitations of the scanner, had to be scanned in two separate scans. The anterior third was scanned at 142 µm and the posterior two-thirds were scanned at 284 µm.

#### Computing equipment and software

The large data sets generated by CT scans require a specialized high performance computer system with significant computational power. This type of system is necessary because of the significant number of voxels contained in each dataset, along with the volume increase resulting from stitching as many as four volumes together. The computer used to analyze these MicroCT data is a Dell T7920 dual 4 GHz (maximum clock speed) CPU (Intel Xeon^®^ Gold 6146) system with 48 threads, 512 GB of ECC memory, and 32 TB of storage, including 6 TB of storage as NVME drives. The software used to work with MicroCT data is 3D Slicer (Federov et al. 2012; Rolfe et al. 2021) version 5.9.0 pre-release.

#### CT data processing and visualisation

**Fig. 1:**
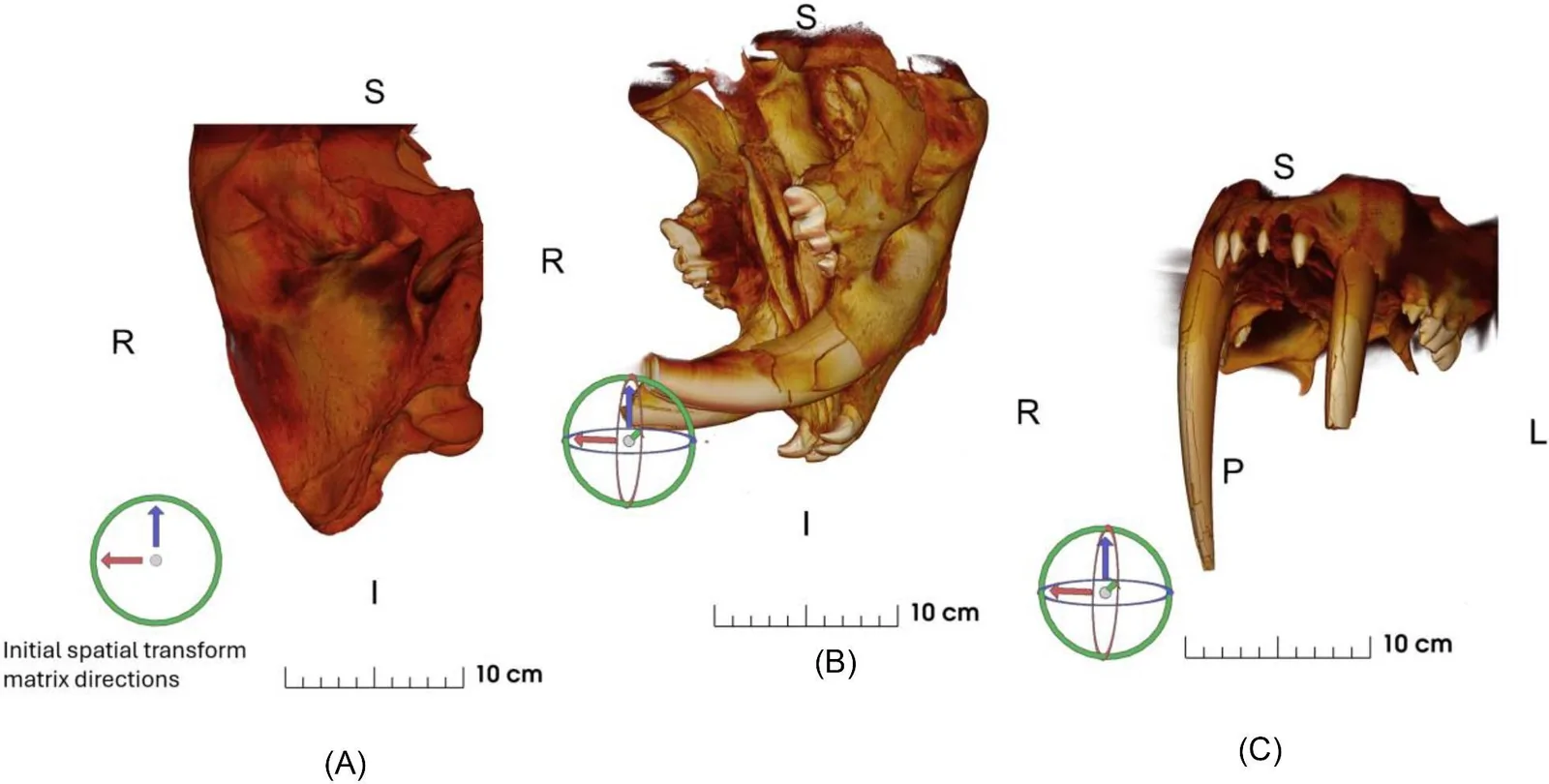
The three MicroCT scans (3D) that make up the LACMH 2001–2 cranium. (A) is the posterior half of the specimen, (B) is the anterior half, and (C) is the maxilla and canines. The three MicroCT scans were limited due to the size of the specimen versus the size the machine could scan at one time. Each scan prior to processing is up to 7 GB compressed. (The three scans were then stitched together digitally in 3D Slicer).

Figure 1 shows the unprocessed 3D images for unmerged LACMH 2001–2. These files are 7 GB of compressed data (up to 70 GB of uncompressed data) each and require the added capacity to manipulate the datasets. The video system features two Nvidia RTX 5000 16 GB cards providing it a total of 32 GB of dedicated video memory, which enables the processing of up to 32 GB of uncompressed file space. If the video file exceeds that, then the graphical processing unit (GPU) needs to be bypassed, and all processing is done in the central processing unit (CPU). This is performed by switching the 3D mode from GPU Ray casting to CPU Ray Casting inside 3D Slicer. The work consists of using the basic MicroCT functions including slices of X-rays and three-dimensional rendering of the X-ray data. These scans can look at both the external and internal aspects of a specimen. Figure 1 is of three MicroCT scans that we stitched together from the LACM 2001–2 cranium. Figure 1(A) is the unmodified posterior half of the specimen, Fig.  1(B) is the unmodified anterior half, and Fig. 1(C) is the maxilla and the canines. Each scan, prior to processing, is greater than 7 GB compressed. Merging these specimens requires a significant amount of memory and a couple of key steps. First, all specimens need to be to be converted from 16-bit to 8-bit by rescaling the intensity. This is due to the shifting machine baseline, e.g., one needs the background “black” to be the same and the bright “white” to be the same. This is gray scale matching for the images. It is also important to learn how to use the transform function to get multiple scans to “stitch” or combine. Prior CT scans did not have this level of resolution, for example, LACM: RLB: R37376, and the previous scans did not require the extra “stitching step.”

The following is a simplified procedure to stitch multiple µCT scans together in 3D Slicer:

- Step one is to import the data from either Dicom files (*.dcm) or Imagestacks (*.tif), 3D Slicer needs SlicerMorph and Sandbox loaded as add on modules, then save in a unique directory.
- Step two would be to remove any voxels by cropping out blank space, i.e., voxels without specimen elements in them and then convert the image to 8-bit gray scale—then save.
- Step three uses percentile rescaling on each 8-bit file to even out the gray scale between the individual scans. This ensures that the background black is the same for all scans of the same skull.
- Step four is to start with a new instance of 3d Slicer and import all the components of the specimen. We will then create individual spatial transforms for each scan and proceed to shift and rotate watching the Slice and 3D views until we get our landmarks on the bones to align.
- Step five is to use the stitch tool in Sandbox to combine the 3D space and save the files.

This is a very simplified description. It would be good to remember that each reduced file is approximately two to three gigabytes.

#### Fitting isolated canine teeth

We will demonstrate how 3D Slicer can be used to determine if a canine that became separated from a cranium belongs to a specimen by placing a canine in a 3D view into a cranium. During this procedure, the visualization of the canine depends on the segmentation feature in 3D Slicer. This “segmentation” tool depends on an underlying scan of the specimen. It assigns a voxel (volume pixel) to every MicroCT scan X-ray voxel with a specific range of image intensities. This step uses the segment editor to highlight the disarticulated canine and then place the canine using a spatial transform to move the canine into the alveolar pocket.

## Results and discussion

This study uses the most complete collection of CT, Micro CT, and NanoCT scans, of *S. fatalis* crania, gathered into a single database, more than what is presently available on MorphoSource. Table 2 Canine and alveolus condition by sample, is a description of the specimens used in this study, including the specimen number and the institution that holds the specimen in its collection, the location where the specimen was scanned, and the current location of the scan. This table also indicates if there is a gap at the apical root and the alveolus damage, if any.

**Table 2:** Canine and alveolus condition by sample.

ID Number	Institution collection	Location scanned	Depository^§^	Canine left gap	Canine right gap	Alveolus damage left	Alveolus damage right
FMNH_P_12417(Fig. 2)	Field Museum	U Chicago—Dept of Organismal Biology and Anatomy	Author—Morphosource	Yes	Yes	No	No
LACMP23-7550(Figs. 3, 4)	Natural History Museum of LA County	USC—Dept of Radiology	Author—Morphosource	No	No	No	No
LACMH 2001–2(Figs. 1, 5, 6, and 7)	Natural History Museum of LA County	USC– Dept of Radiology	Author—Morphosource	No	Yes	No	Yes
LACMH 2001–3(Fig. 8)	Natural History Museum of LA County	USC—Dept of Radiology	Author—Morphosource	No	No	No	No
FMNH_PM_3671(Not shown)	Field Museum	U Chicago—Dept of Organismal Biology and Anatomy	Author—Morphosource	No	No	Missing part of alveolus	Missing part of alveolus
LACM_rlb_R37376(Fig. 8)	Natural History Museum of LA County	University of Texas High-Resolution X-ray Computed Tomography Facility	Morphosource	No	N/A	No	Yes
FMNH_P_12407(Not shown)	Field Museum	U Chicago—Dept of Organismal Biology and Anatomy	Author—Morphosource	N/A	No	N/A	N/A
FMNH_P_12463(Not shown)	Field Museum	U Chicago—Dept of Organismal Biology and Anatomy	Author—Morphosource	N/A	No	N/A	N/A
FAM 14349(Not shown)	American Museum of Natural History	American Museum of Natural History	Morphosource	No	No	No	No
LACMH 6870(Fig. 9)	Natural History Museum of LA County	USC—Dept of Radiology	Author—Morphosource	N/A	N/A	Open suture	Open suture

§ Morphosource is the repository of choice for Natural History Museum of Los Angeles County.

MicroCT scans are an important way to study fossil specimens. This ability comes with some caveats including scanner limitations, the size of scanned files, machine availability, and availability of the specimens. The specific area where the canine fit into the alveolar socket is the focus of our study.

**Fig. 2:**
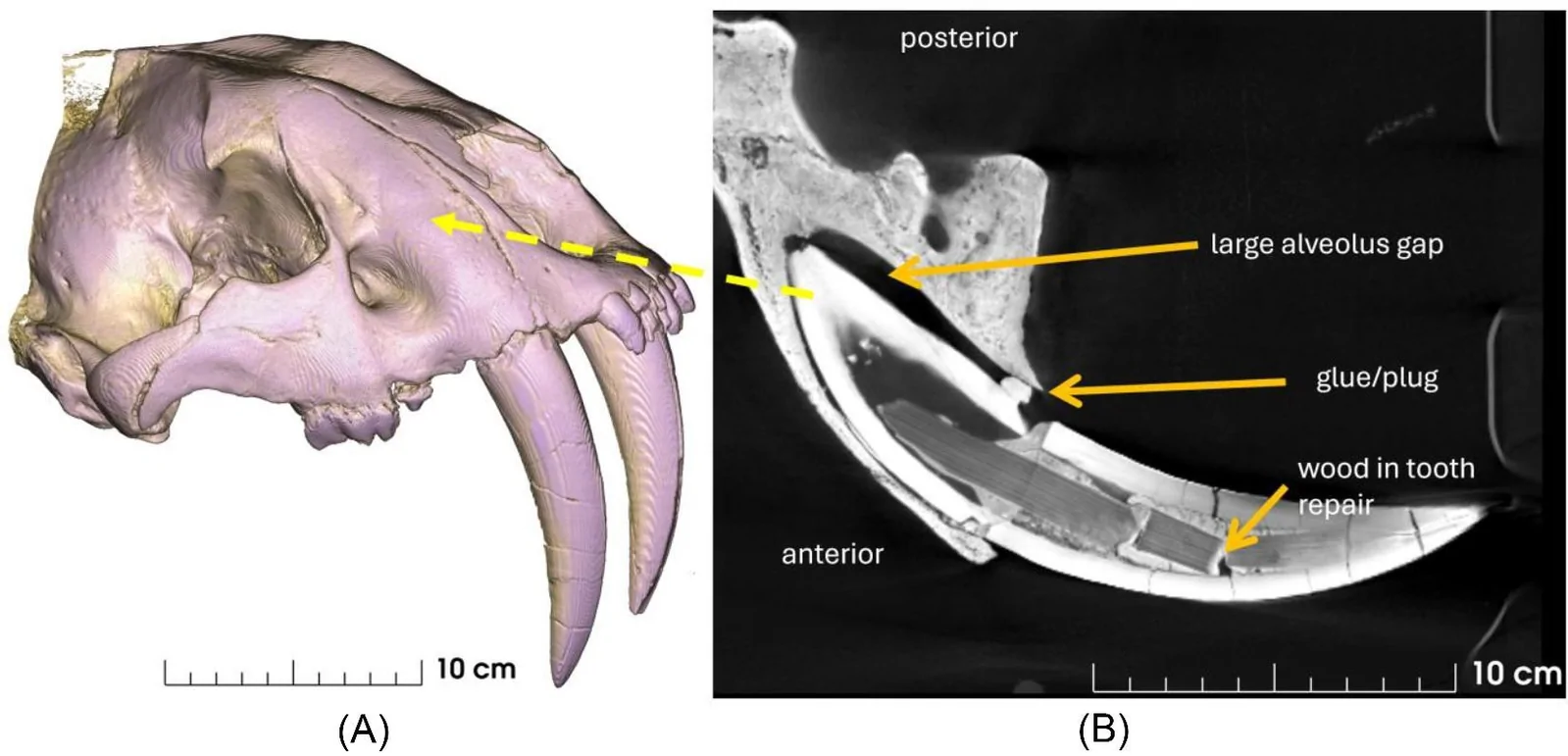
Field Museum FMNH_P_12417. (A) is a lateral view of the right side of the cranium in 3D, taken from the MicroCT scan. (B) is a 16-bit gray scale image of the same specimen in 2D with a close-up of the right canine (rotated to view the arrows better). The arrows identify a large alveolus gap, a glue plug, and wood that was used in the repair of the canine. This specimen is currently on display at the Field Museum.

The FMNH display specimen FMNH_P_12417 is in Fig. 2(A). It was known to have some restoration work but was thought to have its original canines. In Fig. 2(B) is a single 2D slice. The arrow points to a gap between the apical tooth root and the apex of the alveolar socket of the left canine. This image reveals that the canine is too small for the alveolus. One unexpected outcome from this MicroCT scan is that the glue/plug and the wood that has been inserted into the canine was not visible externally but appeared only in the MicroCT data. The wood in the canine, placed there during preparation, usually called “restored for display” illuminates how restorations can affect perceptions.

This reconstruction was done with canines that do not fit properly. These types of reconstruction often shape public perceptions, but they are not necessarily accurate. For example, this alteration makes the canine extend longer than it would if fully seated in the alveolus. The use of non-associated canines distorts the perceptions of *S. fatalis* and can affect the approach the scientific community can take during analysis.

**Fig. 3:**
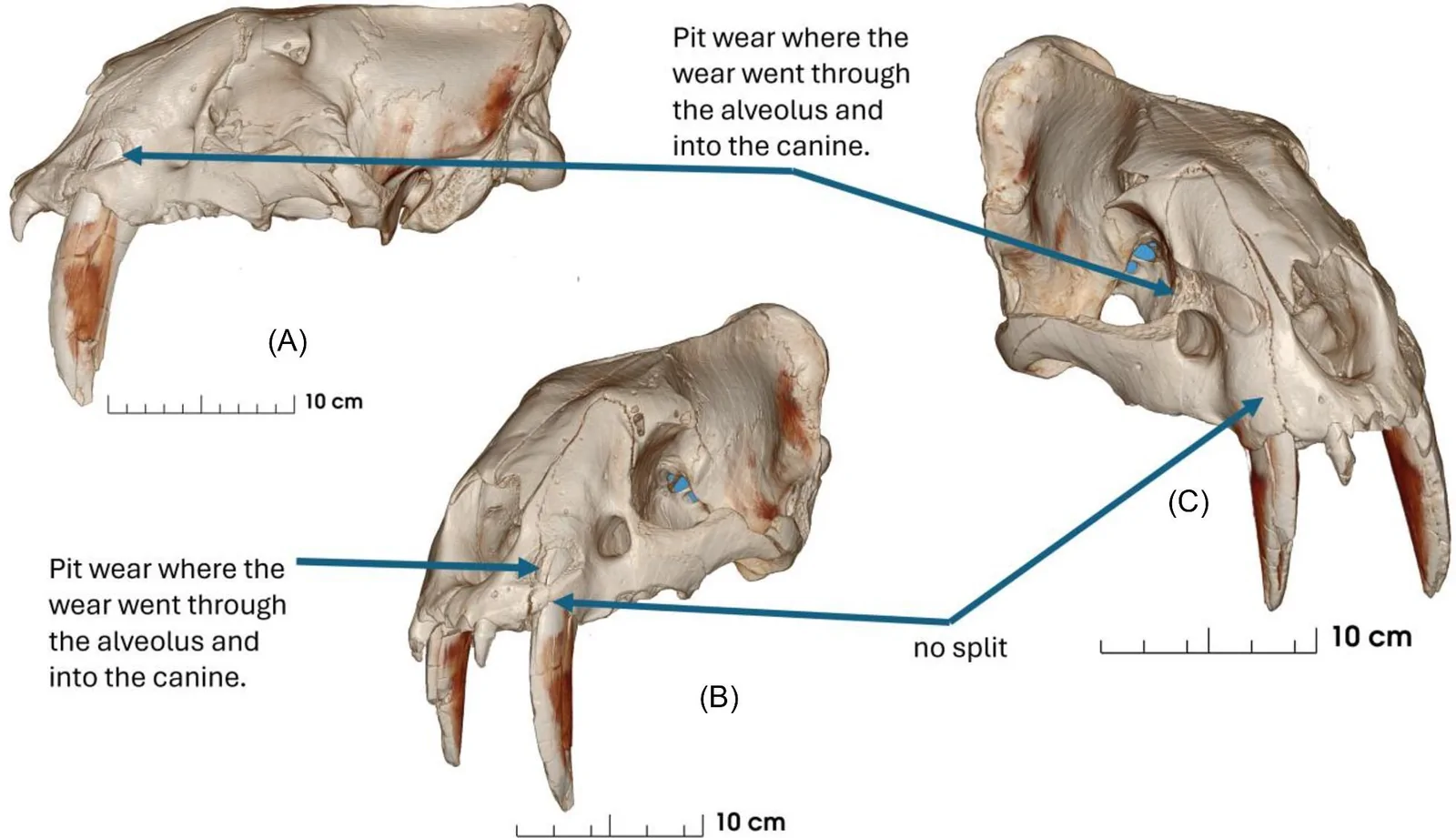
LACMP23-7550 3D MicroCT scan of the exterior of the skull in 3D. (A) is a MicroCT scan of the exterior of the cranium (left lateral view). The arrows in (B) and (C) identify pit wear. Additionally, the arrows on (B) and (C) point to the sutures.

Specimen LACMP23-7550 was discovered in 2008 in the Project 23 excavation described by Portner (2025) and was made available to us during one of our visits to the LBTPM collection on November 7, 2021. It is also one of the best conserved and traceable specimens. Figure 3 is a 3D rendering of a MicroCT scan of the exterior of the skull. This is the first time that this specimen has been scanned by MicroCT. This cranium has two canines that are fully seated in the alveoli.

In Fig. 3, the exterior of the skull with several areas of pit wear are visible (Miller 1968; Spencer and Van Valkenburgh 2003). Pit wear is damage caused to the bones while they were immersed in the asphalt in the tar pit. The asphalt contains dirt, rocks, branches, bones of this specimen, and other specimens. The asphalt moves when gas is released through it. Over time, the movement causes these items in the asphalt to rub spots in the bones causing a unique type of damage to the bones. This gas release moves the liquid asphalt, and this is still occurring today at LBTPM. Pit wear is well known for collections in asphalt pits but is not well known in other fossil sites that do not contain asphalt seeps.

The pit wear on LACMP23-7550 was the first indication that the canines in this specimen were fully seated in the alveolar pocket. Figure 3(A) is a left lateral view. The arrows in Figs 3B) and 3C) point out pit wear damage (Miller 1968; Spencer and Van Valkenburgh 2003). In this figure, the damage goes through the bone, into the enamel, and continues into the dentine of the canine. The arrows in Fig. 3(B and C) illustrates the sutures. We observed no additional damage to the alveolus. The MicroCT scans can not only render the exterior bones in 3D, but they also show the interior of a specimen, including bones and teeth in 2D.

**Fig. 4:**
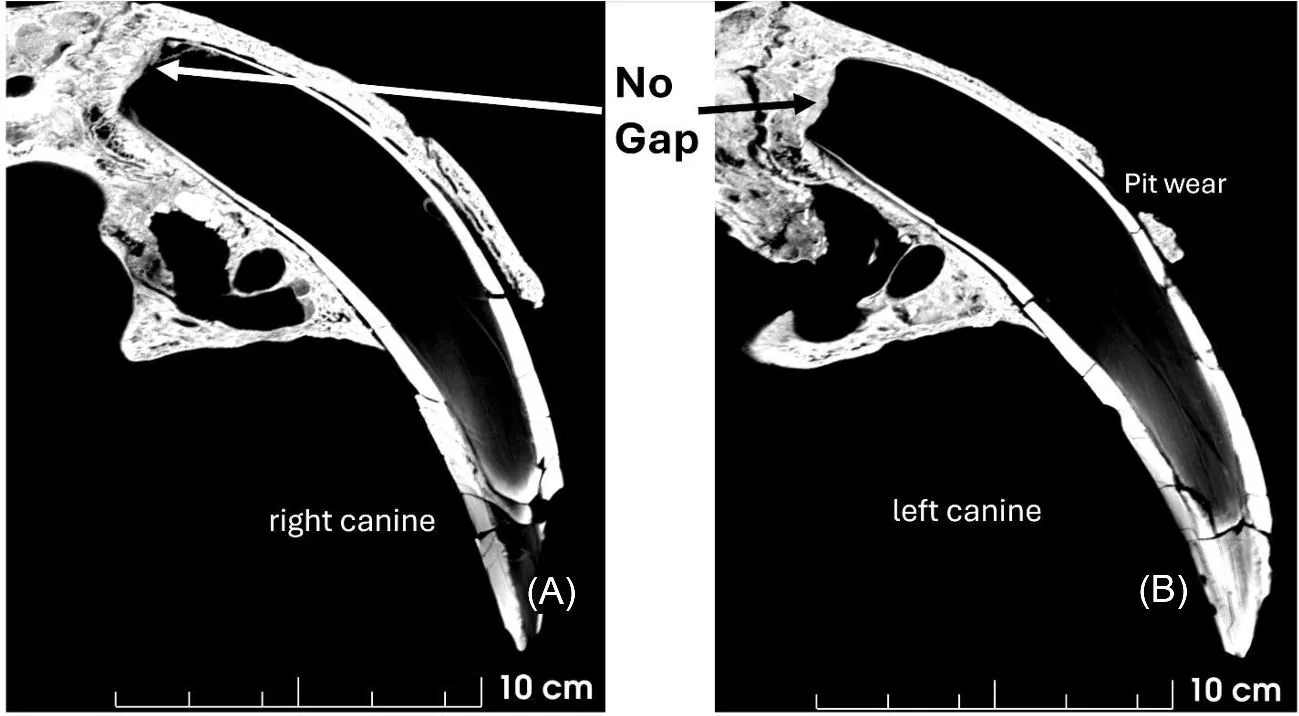
Canine placement in LACMP23-7550. Using 2D MicroCT scans (A) is the right canine sliced through root and (B) is the left canine root slice. Both canines fit fully into the alveoli without a gap at the apical root. Both images are 16-bit gray scale.

Figure 4(A) is the 2D slice of the right lateral view of LACMP23-7550. Figure 4(B) is the left lateral view of the same specimen with the sutures visible at the alveolar socket. These sutures in Fig. 4 are not split. The MicroCT 2D slice in Fig. 4(A and B) confirms the placement of the canines and ensures that both the right and left canines are fully seated in the alveolar socket. We want to point out that in this specimen both canines fit fully into the alveolus without a gap at the apical root. The teeth have some fracturing but have been conserved *in situ*.

**Fig. 5:**
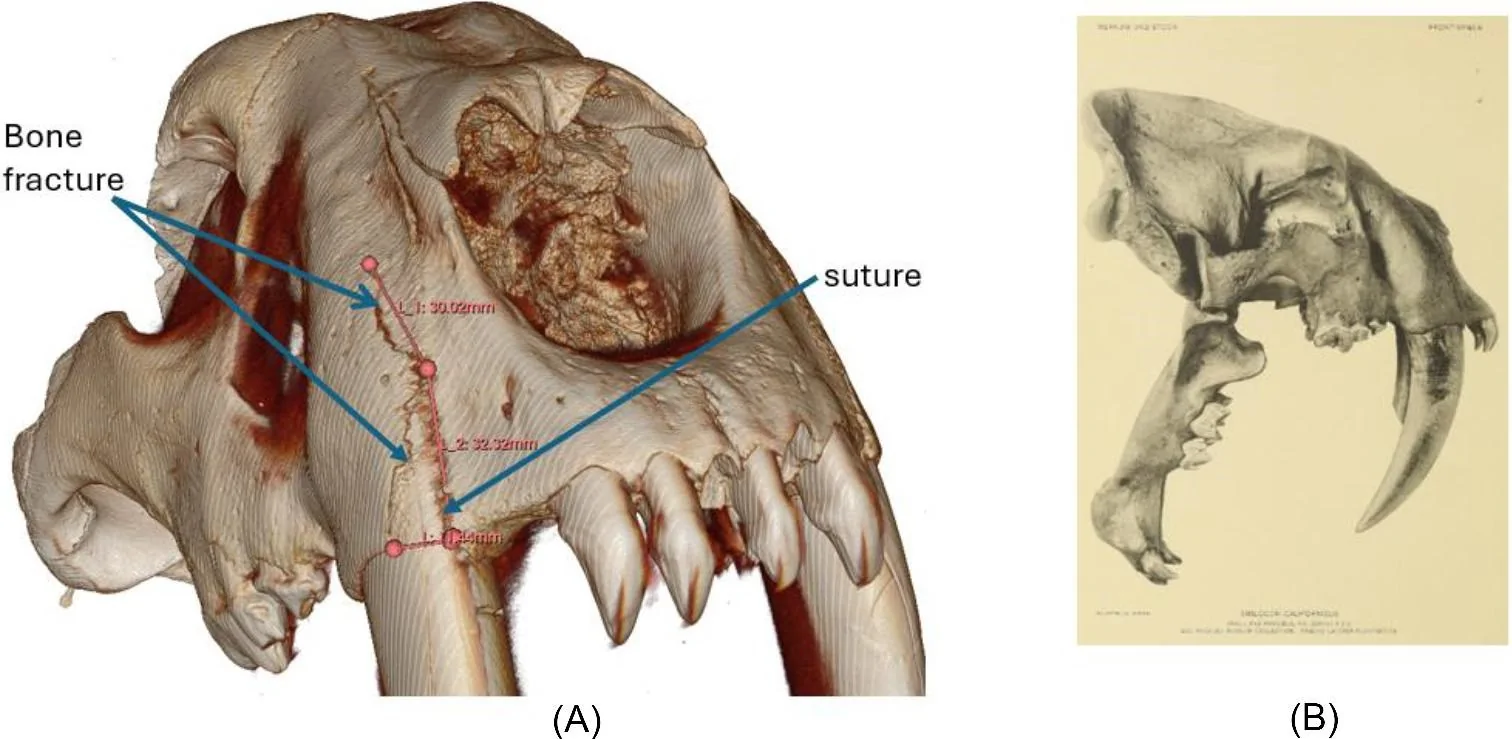
Two different images of LACM 2001–2. (A) is a 3D MicroCT rendering demonstrating a 62.3 mm split in the right alveolus along the suture. The split is 11.4 mm wide at the crest. This fracture most likely initiated in the alveolar bone and propagated dorsally. Figure (B) is the title figure of “Felidae Rancho La Brea” (Merriam and Stock 1932). *Note*: The image on the right is from a personal copy of The Felidae of Rancho La Brea by Merriam and Stock and is therefore dated older than the copyright time limit.

One of the most famous specimens in this study is LACMH 2001–2. Merriam and Stock (Merriam and Stock 1932), Akersten (Akersten 1985), and others (Tejeda and Shaw 1984; Duckler 1997.; Christiansen and Harris 2012; Wheeler 2018; Deutsch et al. 2025) have studied this skull, making it important to include this specimen in our study. The MicroCT scans gave us the ability to look internally and to measure various areas of a specimen. In Fig. 5(A) the sutures and a fractured alveolus can be seen. There is a 62.3 mm split in the right alveolus. Additionally, the split is 11.4 mm wide at the alveolar crest, and a piece of the bone has fallen off. Figure 5(B) is from Merriam and Stock’s “Felidae of Rancho La Brea” (Merriam and Stock 1932) and is one of the more famous images of this felid.

**Fig. 6:**
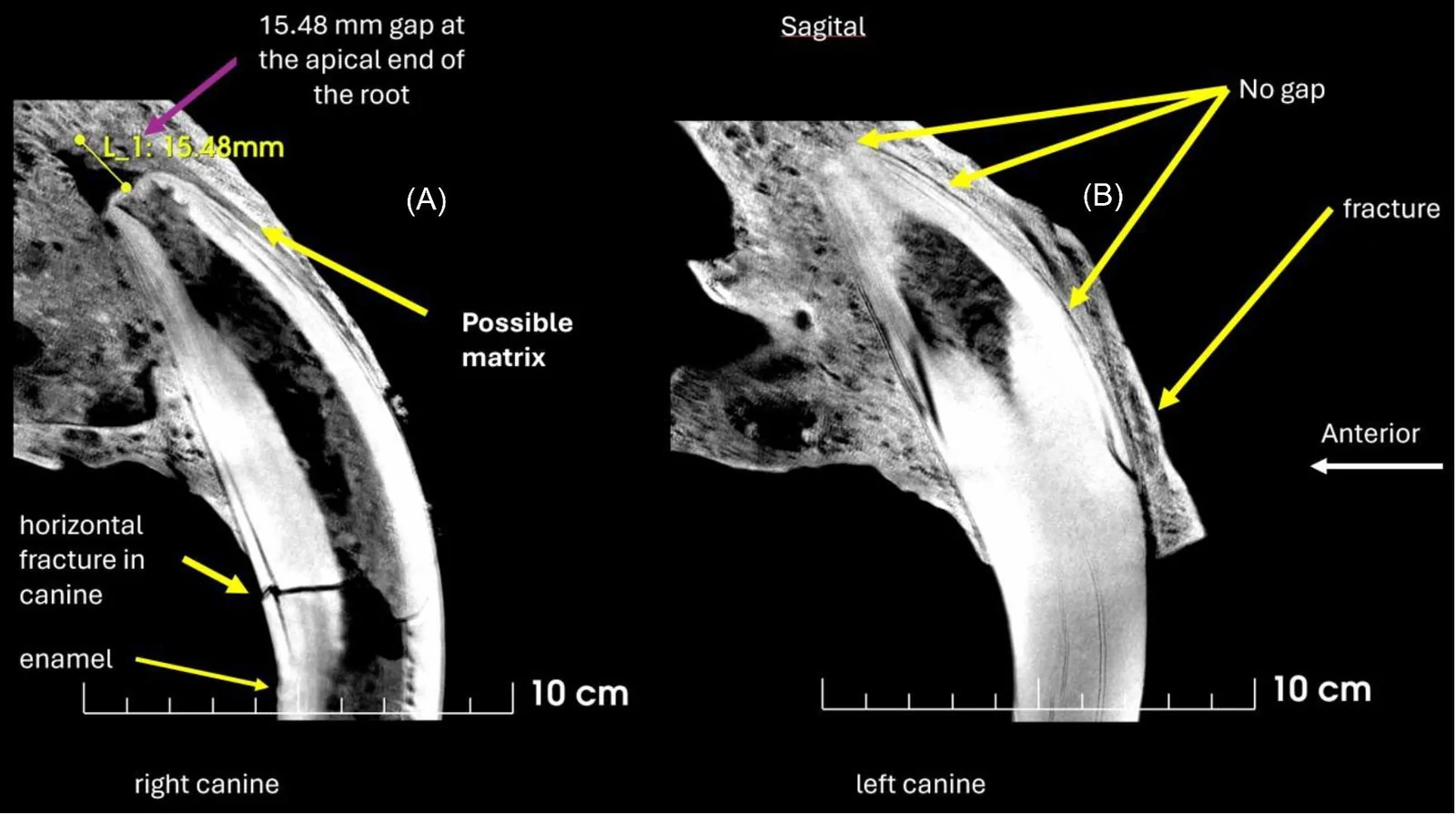
Tooth placement in the alveolus of LACM 2001–2. (A) and (B) are 2D MicroCT slices of the right and left canine, respectively. (A) is a16-bit gray scale of the right canine showing a misfit of the right canine to the alveolus and signs of considerable mechanical force along with the presence of matrix in the alveolus. (B) is the left canine, that has a proper fit of the canine to the alveolus. The left canine has been broken so that a length measurement could not be taken. There is no sign of force applied to the canine.

The image in Fig. 6(A) is also of LACM 2001–2 with the apex of the root at the lower left. The right alveolus is fractured and reveals that the right canine is misaligned in the alveolus, possibly due to mechanical force, i.e., a force applied by external methods such as a person forcing the canine back in. The arrows in Fig. 6(A) point to the broken canine, and a gap of 15.4 mm at the apical end of the root. The gap at the end of the canine appears to contain a considerable amount of matrix, as pointed out in the image. The MicroCT scans for this specimen identify the questionable provenance of the right canine. Figure 6(B) is in the same orientation. The left canine in Fig. 6(B) contains a proper fit of the canine to the alveolus. There is no sign of force applied to the canine. An alternate hypothesis might be that the single-rooted canine might have slipped while it was in the slowly moving asphalt. This thought is that the matrix could have been infilled over time. Regarding specimen LACMH 2001–2, it appears to have matrix in the alveolar gap, the canine would have to have slipped a significant amount and then be replaced. The matrix would then be visible around the canine as well as in the alveolar gap. We do not see this occurring in the MicroCT scan. It is also possible that the single-rooted canine slipped out in the asphalt seep and due to bone shrinkage, the canine could not be properly reinserted. Bone shrinkage has been studied by Lees (2005), Lievers et al. (2007), Bertrand and Oxenham (2015), Ellingham and Sandholzer (2020), Walden et al. (2021), and others.

**Fig. 7:**
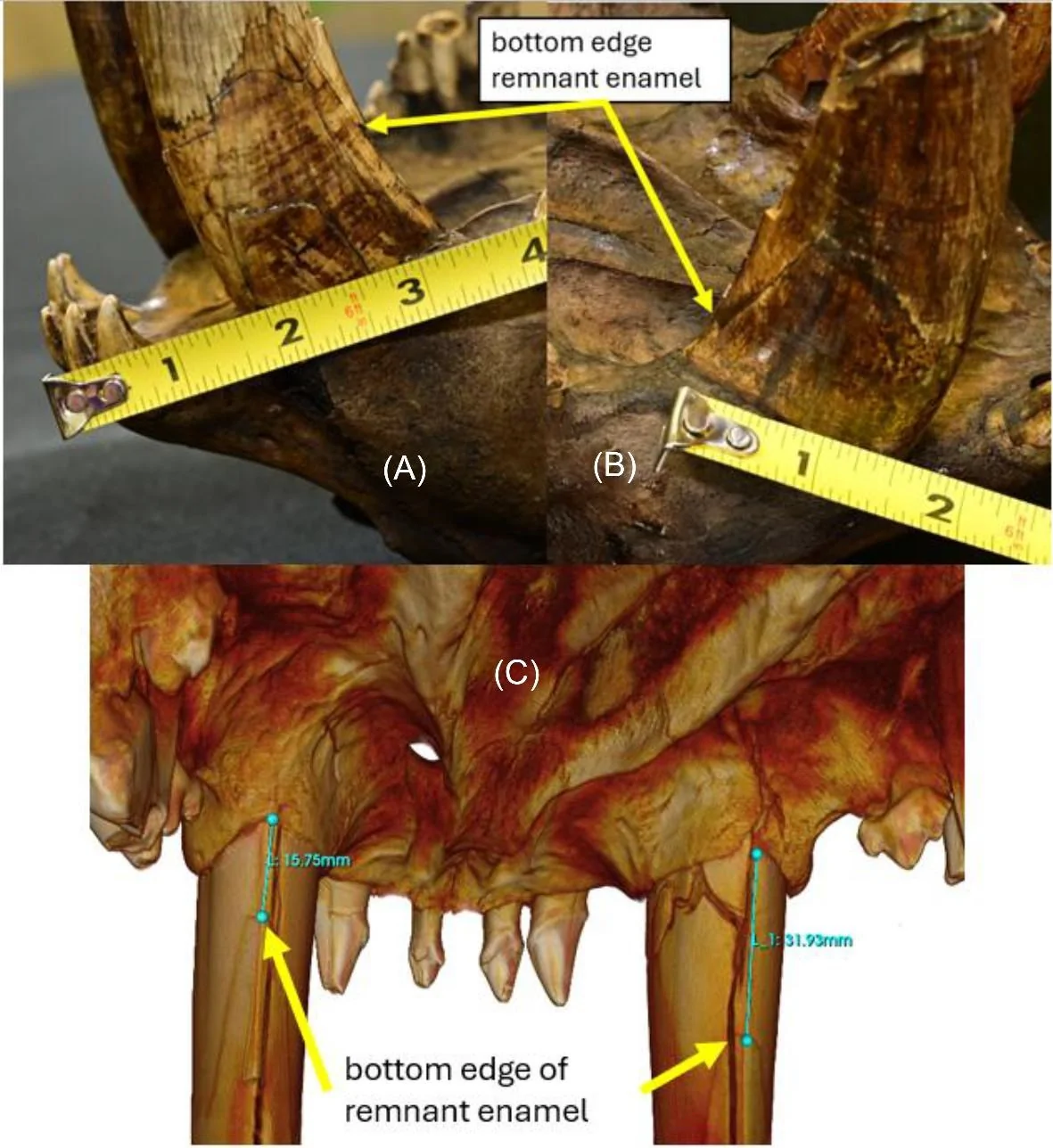
This figure, LACM 2001–2, reveals that external indicators show the right canine was not seated properly. (A) is a photograph of the posterior edge of the right canine’s remaining enamel. (B) is a photograph of the posterior edge of the left canine’s remaining enamel. (C) is a 3D rendered MicroCT scan image of the posterior edge of both canines’ remaining enamel. This image identifies the measured mismatch of the remnant enamel is 15–16 mm. Photographs in (A) and (B) were taken by the author Misty S. Haji-Sheikh. (1 inch = 25.4 mm).

When inspecting LACM 2001–2’s canines in 2020, we noted that the remnant enamel (posterior serrated edge) was mismatched with respect to their alveoli. Akersten notes that the enamel, on the posterior edge of the canines stop at the gumline (Akersten 1985). This is detailed in Fig. 7(A and B) and includes both the photographic and MicroCT evidence of a 15–16 mm mismatch. Figure 7(C) is a photograph from LBTPM taken by the authors and annotated showing that there is a difference from the bottom of the enamel to the original top of the crown. This figure illustrates the MicroCT evidence of a 15–16 mm mismatch in the remnant enamel. The differential external measurement was approximately the same as the measured gap at the apex of the canine.

**Fig. 8:**
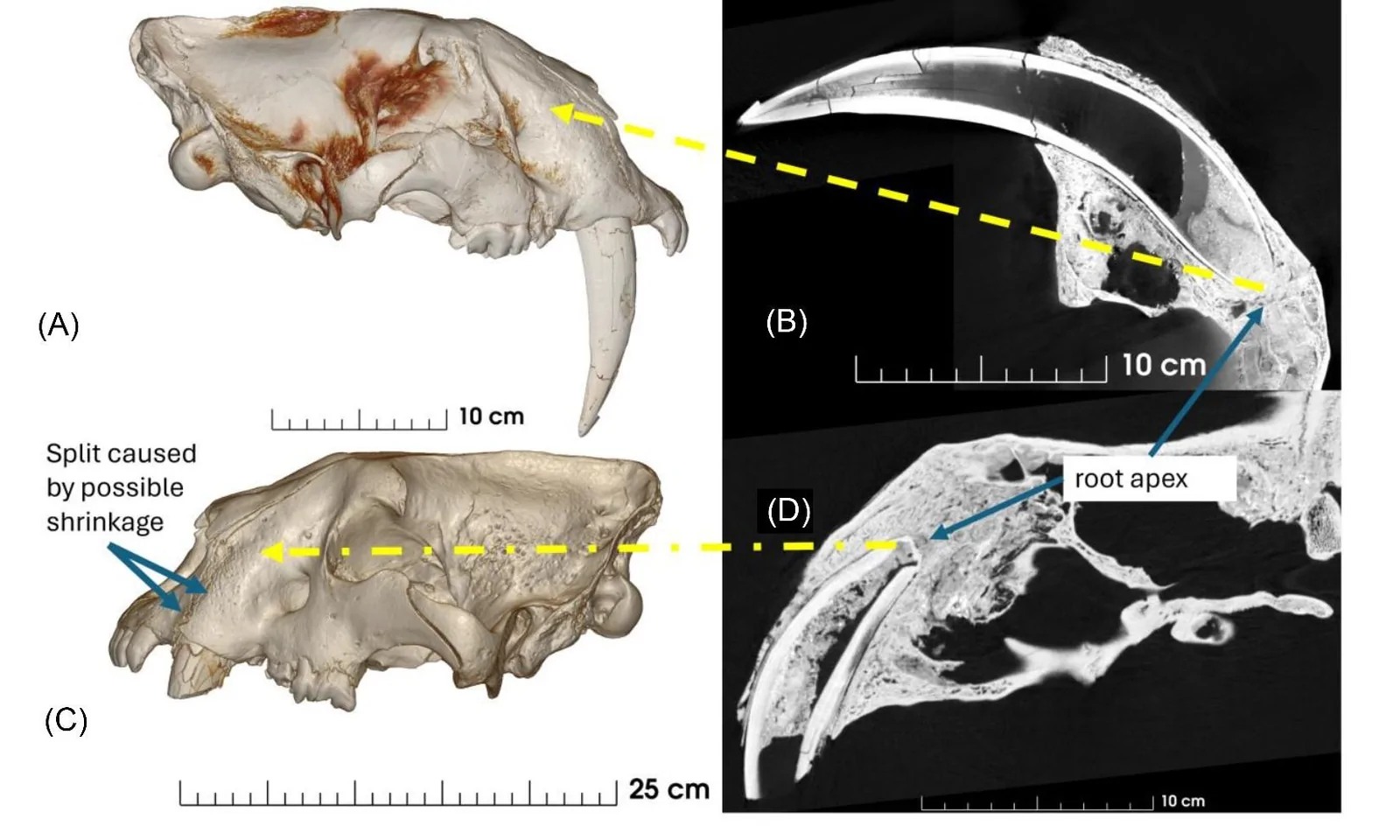
MicroCT scans. (A) and (B) are LACM 2001–3 and (C) and (D) are LACM_rlb_R37376. (A) is an external 3D view of the cranium with the arrow pointing to the suture with no splits. (B) is a 2D slice of the right canine with the apical end of the root to the lower right. The arrow indicates no gap. (C) is a 3D scan with the incisors to the left. (D) is a 2D slice of the left canine root, fully seated. This image was rotated to see the arrow.

Figure 8 is LACM 2001–3 and LACM_rlb_R37376. Figure 8(A) is a 3D view of LACM 2001–3 with one complete canine and one broken canine. The specimen is in good shape. The external view of the skull presents the suture with no splits. Figure 8(B) is a 2D slice of the right canine with the apical end of the root to the lower right. The root is fully seated in Fig. 8(B). The arrow highlights no gap. The cranium was hit by a pickax, but this is not visible on the image. The specimen in Fig. 8(C) is LACM_rlb_R37376 and has been used in multiple studies, including studies by B. Figueirido, S. Tucker, and S. Lautenschlager (Figueirido et al. 2018; Figueirido et al. n.d.; Janis et al. 2020). This specimen was initially scanned for Van Valkenburgh in 2003 (Van Valkenburgh 2024). This specimen only has one partial canine, so it would be difficult to use for bite studies. We can still determine whether the canine root is original to the specimen with the MicroCT scan. The canine would not be fully seated in the alveolus if it had become dislodged, but this canine is fully seated. The suture has widened postmortem from what it would have been antemortem. Since the canine and root appear to be intact at the apex, the split then might be caused by some bone shrinkage. One of the possible sources of shrinkage could be due to the moisture leaving the bones (e.g., desiccating) (Lees 2005; Lievers et al. 2007; Bertrand and Oxenham 2015; Ellingham and Sandholzer 2020; Walden et al. 2021). Figure 8(D) is a 2D slice that displays that this root is fully seated in the alveolus. Splitting appears to have happened postmortem. The arrow points to the apical root of the canine, identifying no gap.

**Fig. 9:**
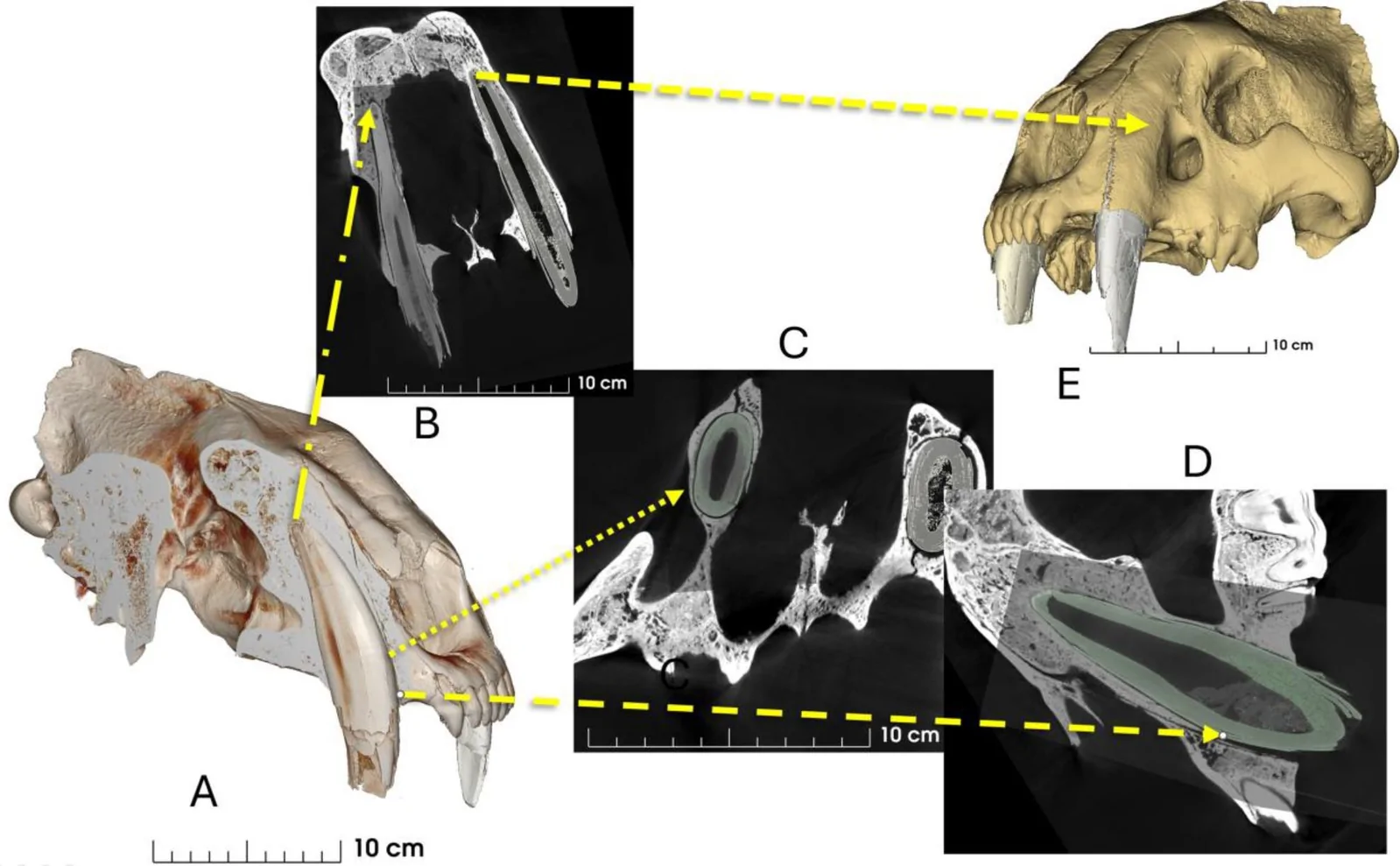
Virtual insertion of the right canine into the adult specimen using 3D Slicer on LACMHC 6870. These images are in chronological order of the process. ( A) and (E) are MicroCT 3D images while (B), (C), and (D) are 2D MicroCT slices. (A) is a lateral view of the right canine with the canine fully inserted into the alveolus. (B) shows the apical root end of the fully inserted canine (shaded). (C) is a cross-section slice of the canine (shaded) . (D) is a lateral slice of the canine fully inserted into the alveolus (shaded). (E) is an external view of the left side of the skull with virtually inserted canines.

One of the interesting uses of 3D Slicer is to be able to virtually combine multiple MicroCT scans to visualize how a disarticulated specimen that has all the components will look when recombined. A reconstructed image of LACMHC 6870 is shown in Fig. 9. When a canine has become dislodged, it is important to ascertain if a particular canine is associated with a specific cranium. Figure 9 has two dislodged canines. This specimen and its two assumed associated canines are studied here to determine if the canines are associated with LACMHC 6870. To test this hypothesis, we concentrated on the right canine demonstrating that the canine fits into its respective alveolus. These images are in chronological order of the process. Figure 9(A) is the three-dimensional rendering of LACMHC 6870 with its right canine digitally inserted. Figure 9B–D is 2D slices of the right canine *in situ* in three axis (green). The apical root is fully inserted in all three views. Figure 9(E) is a 3D external view of skull with virtually inserted canines using MicroCT. These canines clearly fit.

In addition to the figures above, we evaluated the additional specimens FMNH_PM_3671, FMNH_P_12407, FMNH_P_12463, and FAM 14349 that are not shown. FMNH_PM_3671 is a well-known specimen used by Radinsky (1975) for creating an endocranial cast. It was important to scan the specimens that have been used extensively in the past to gain new information. By observation of the scans these canines are completely seated in the alveoli with no gap. The basioccipital suture and open root foramen indicate that this specimen is probably a pre-adult specimen. It is also important to note that broken specimens have valuable information. This specimen was broken, and some of the palate is missing; nevertheless, this specimen highlights the importance of studying partial specimens. Two more broken or damaged specimens FMNH_P_12407 (right maxilla) and FMNH_P_12463 (partial skull, anterior) were scanned and revealed that their canines are well seated.

The AMNH specimen FAM 14349 is available on MorphoSource, with permission from AMNH, but with significantly lower resolution than our new scans. The canine roots for FAM 14349 were observed to be well seated with both canines broken and the alveolus has no external splitting. Inspection of the MicroCT data

demonstrates how strong the root connection can be by showing no separation in the alveolus/tooth interface even though the canines were mechanically damaged. The arrow points to the apex indicating that there is no gap.

**Fig. 10:**
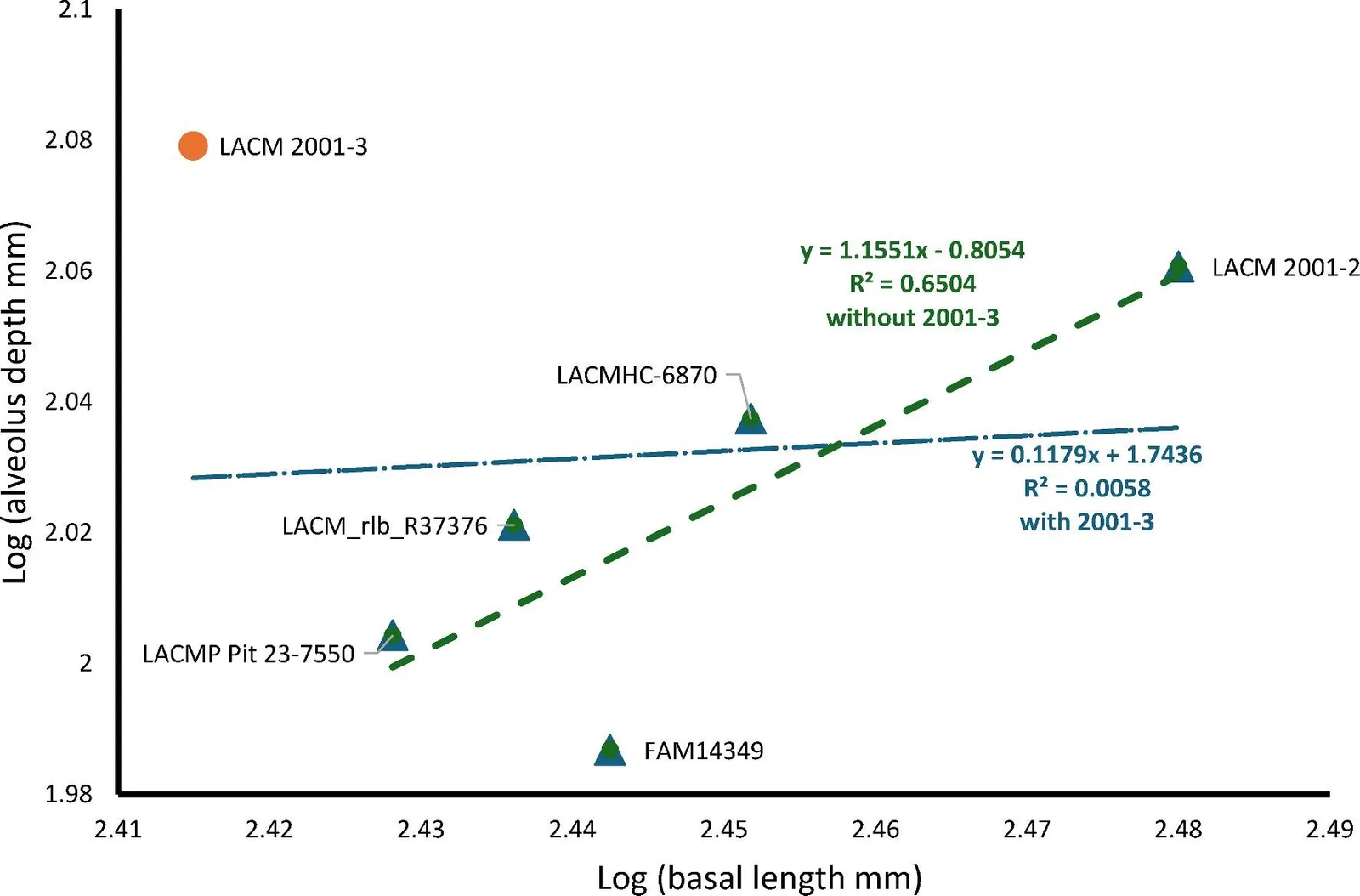
Alveolus depth vs. basal length. There is no direct relationship between basal length and the alveolus depth in these specimens most likely due to the age range of the specimen (Akersten 1985), relatively small sample size, and small statistical range of alveolus depths. Basal length is measured from the center of the posterior edge of the incisor alveolus to the foramen notch. Average depth is 107.8 mm with a standard deviation of 7.9 mm.

Figure 10 is the linear regression for a small data set of six specimens. If we include LACM 2001–3, then this regression set is poorly correlated. The *R*^2^ becomes very small. When we remove LACM 2001–3, the five data points are well correlated with a *R*^2^ of 0.65. At this point, we have no statistical reason to remove LACM 2001–3.

## Conclusions

This study began with noticing that in adult specimens the enamel to alveolus opening would be the same for both alveoli, and the canine serrations would stop at the gum line. At that time, there were no studies on the canine root depth or the alveoli socket. The external scans cannot identify the alveolar gap at the apical root end if both teeth have enamel stopping at the same place. And the possibility that the canines were fully seated in the alveolus or that slippage or handling issues could have occurred. The MicroCT scans also allow us to determine if the canines belong to the skull they are with, and even if isolated canines that belong to an associated skull.

To determine the canine length and that the canines are properly seated in the alveolar socket, it was necessary to go beyond external scans and external measurements. This is because it is not possible to see the canine root apex inside the skull. The improper canine length would invalidate a study based on external scans and/or external measurements.

We have demonstrated that we can use CT and MicroCT scans to determine the alveolar condition of various Smilodon fatalis crania and we summarize the findings in Table 1. Of the four specimens with complete canines, two of the specimens were “restored for display.” This includes one of the most iconic examples of *S. fatalis*, beautifully rendered in “The Felidae of Rancho La Brea” (LACMH 2001–2) (Merriam and Stock 1932). We demonstrate that surface clues can be used to detect whether the specimens have been “restored for display” (Simpson 2025), and MicroCT can verify these clues. One of the key indicators is the presence and position of the enamel on the teeth. Since the *S. fatalis* specimens have serrated canines, the location of the serrated edge of the canine with respect to the alveolar crest can be an important observation.

We successfully digitally inserted a canine in its respective alveolus. 3D Slicer allows us to verify whether a canine belongs to a certain specimen by allowing us the ability to virtually insert the right canine into a *S. fatalis* (LACMH 6870) specimen, and additionally, we did the same for the left canine. This may be the first time 3D Slicer has been used on *S. fatalis* to attempt to determine if a canine belongs to a certain skull.

We used 10 specimens (20 possible alveoli). Twelve of the 20 possible alveoli show no gap between the canine and the apical root end; however, three had alveolar gaps. The rest of the alveoli were unusable. This is an important finding because this feature cannot be observed from the exterior of the cranium. The importance of the gap at the apical root end is that it makes the crown of the canine appear longer than the actual *in-vivo* length would be. This can have implications for downstream functional modeling approaches. For example, Finite Element simulations of the saber-tooth bites (e.g., see Wroe et al. 2013 and single tooth see Tseng 2025), where changes in canine length will result in different bite strength and the mechanical strength of the canine. Or in gape analyses (for example, see Deutsch et al. 2025), where changes in canine length will impact the effective gape angle (angle between the tips of the lower and upper canines). We recommend implementing CT scanning or using only CT scanned specimens to determine canine seating and provenance in such studies in the future.

## Supplementary Material

obag014_Supplemental_File

## Data Availability

All data used in this study are defined in the manuscript or available from the author on reasonable request, as there is no Supporting Information or a public data repository outside of MorphoSource. The MicroCT scans will be released in Open Source in coordination with Field Museum of Natural History and La Brea Tar Pits and Museum.
