# Correction to: *Smilodon fatalis* Canine and Alveolus Junction Gap: Using MicroCT Scans and 3D Slicer

**DOI:** 10.1093/iob/obag058

**Published:** 2026-10-08

This is a **correction** to:

M S Haji-Sheikh, M J Haji-Sheikh, V L Naples, *Smilodon fatalis* Canine and Alveolus Junction Gap: Using MicroCT Scans and 3D Slicer, *Integrative Organismal Biology*, Volume 8, Issue 1, 2026, obag014, https://doi.org/10.1093/iob/obag014

In the originally published version of this manuscript, the second and third paragraphs of the ‘Results and discussion’ section were inadvertently duplicated.

The duplicate paragraphs has been removed from the paper.

