# Supplementary material for: Smilodon fatalis Canine and Alveolus Junction Gap: Using MicroCT Scans and 3D Slicer
Source: Integr Org Biol. 2026 Apr 13;8(1):obag014. doi: 10.1093/iob/obag014 (PMC13136899; doi:10.1093/iob/obag014)
Supplement: obag014_Supplemental_File [file obag014_supplemental_file.pdf]

Supplemental FMNH_P_12418. This sample was used in Wroe et al. (Wroe, Chamoli, Parr, Clausen, Ridgely & Witmer, 2013) for the basis of a Smilodon fatalis FEA model. The paper explicitly states one complete canine that they mirrored for the missing canine in their FEA. The Field Museum states that the canine has a different specimen number and the other root is a sculpted root. This is a completely unassociated canine which has been chiseled to fit in the alveolus for show.

From Wroe et al. (Wroe et al., 2013)

"Specimens of both *T. atrox* and *S. fatalis* each retained a single complete upper canine including tooth roots. A complete upper left canine (*T. atrox*) and complete upper right canine (*S. fatalis*), including the tooth roots, were segmented out from the remainder of the crania for both. These were mirrored in Mimics (vers. 13.02) to provide complete upper canines on the opposing sides."

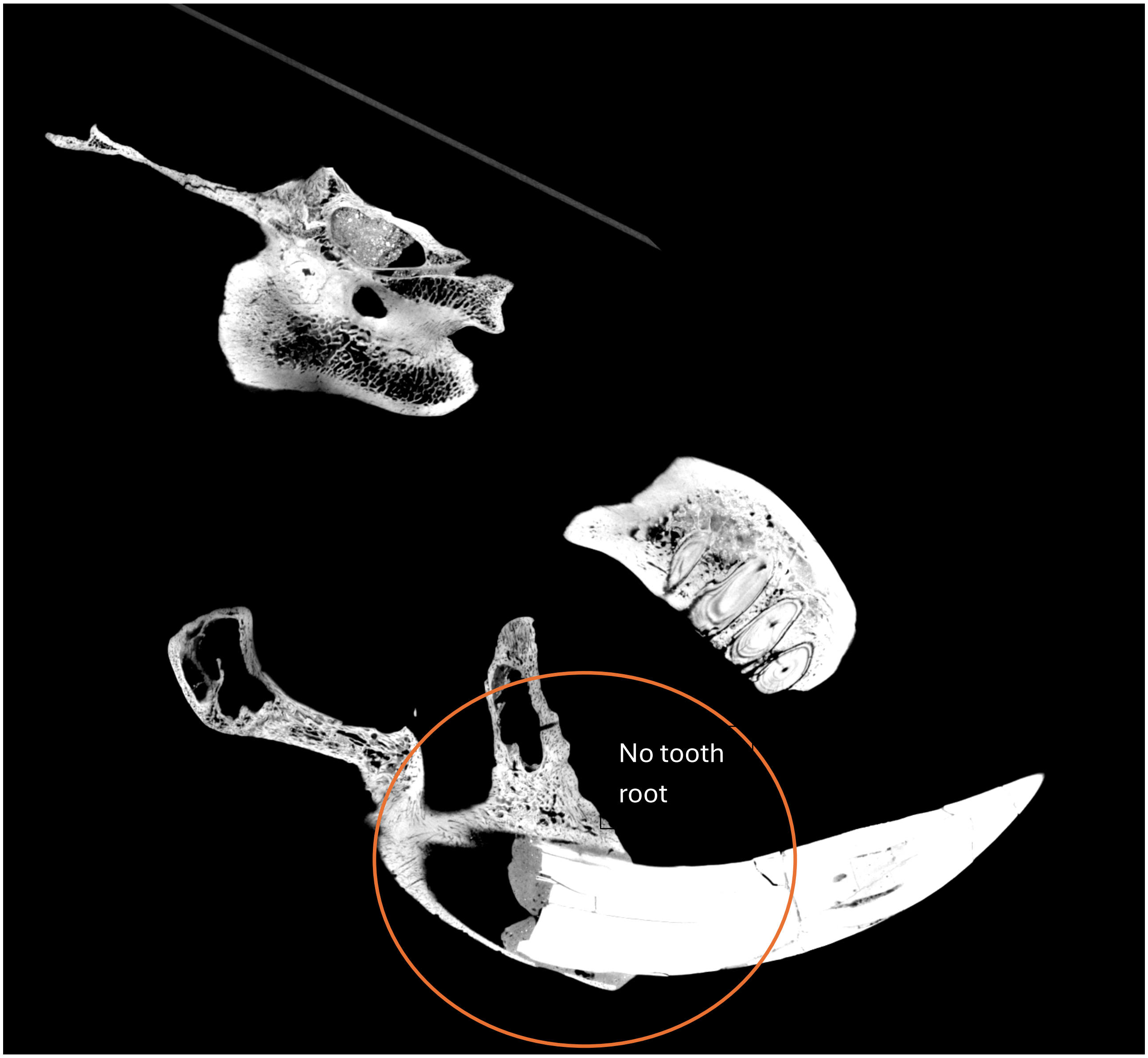

**Date scanned**	02/11/2026
**Specimen number**	FMNH P 12418
**Specimen name**	Smilodon fatalis
**Description**	Smilodon skull with metal fixture inclusions
**Operator**	SMSmith
**PI/Requester**	Ken Angielczyk
**Machine**	NSI X5000
**Tube**	225
**Voltage (kV)**	170
**Current (uA)**	540
**Focal spot size (um)**	91.8
**Recon voxel size (um)**	94.332
**Framerate (FPS)**	2
**Frames averaged**	4
**Filter**	0.060" Cu (wheel)
**Gain Mode**	3
**Type**	continuous

References:

Wroe S, Chamoli U, Parr WC, Clausen P, Ridgely R, Witmer L. 2013. Comparative Biomechanical Modeling of Metatherian and Placental Saber-Tooths: A Different Kind of Bite for an Extreme Pouched Predator. *PLOS One* **8:** e66888.
